# “Enhancing equitable impact: ethical, legal, and sustainable approaches in short-term surgical outreach for global child health”

**DOI:** 10.3389/fped.2024.1398432

**Published:** 2025-01-10

**Authors:** Zoe Blumenthal, Walid A. Farhat, Kelly McQueen

**Affiliations:** ^1^ Department of Urology, University of Wisconsin Foundation, Madison, WI, United States; ^2^ Department of Anesthesiology, University of Wisconsin Foundation, Madison, WI, United States

**Keywords:** global health, outreach, pediatrics, missions, equity

## Abstract

Global health prioritizes improving health and achieving equity in health for all people worldwide. It encompasses a wide range of efforts, including disease prevention and treatment, health promotion, healthcare delivery, and addressing health disparities across borders. Short-term medical and surgical missions often contribute to the global health landscape, especially in low and lower-middle income countries. These programs aim to provide healthcare services to underserved populations and-often face challenges related to governance, ethical boundaries, and legal dimensions. Without adequate oversight and accountability, short-term medical and surgical missions may inadvertently perpetuate harmful practices, negatively impact the native healthcare system, and/or fail to address the long-term health needs of the communities they serve. The impact of short-term surgical trips has raised concerns regarding the need for capacity building initiatives, as well as the ethical and legal aspects of short-term medical and surgical missions. Short term surgical aid has a long history and has likely positively impacted the lives of children and adults with no other option for surgical, especially complex surgical care. These same short-term interventions have also been appropriately criticized for a lack of continuity of care, limited focus on training and education of local providers and capacity building within the local health systems, and perpetuation of power imbalances and neocolonialism. In response, there is an increasing call for a more comprehensive approach that incorporates capacity building and establish robust frameworks that ensure quality improvement, outcomes analysis, ethical conduct, sustainability, and equitable impact of short-term medical and surgical missions within global health. Child health is a critical concern, especially in less developed countries where almost half the population is under 20 years old. Pediatric surgical conditions have a significant impact on child health, and integrating surgical care with global health initiatives can effectively address important child health goals. Global surgery and global pediatric surgery aim to improve health outcomes and achieve equity in surgical care for underserved populations. Pediatric surgical conditions encompass various diseases, with a substantial portion requiring time-sensitive interventions. Establishing sustainable pediatric surgical capabilities within local health systems, including governance, alignment with health priorities, and effective leadership, is crucial. Selecting and supporting individuals for training and ensuring timely access to quality specialist advice are essential for achieving positive clinical outcomes. This review examines existing recommendations for ensuring the sustainable benefit of short-term medical missions, with a specific focus on surgical outreach trips. It highlights the need for standardization and emphasizes the importance of considering the legal and ethical dimensions in guiding these missions. Key aspects include the promotion of local leadership, cultural contextualization, and field-testing of guidelines. By incorporating these elements, medical missions can strive to achieve quality improvement, adhere to ethical principles, and operate within legal frameworks, thereby maximizing their impact and contributing to global health endeavors.

## Introduction

Access to surgical care, especially emergency services, remains a significant challenge for 2/3 of the worlds populations particularly those in rural areas and in low- and middle-income countries (LMICs) (1, 2). The scarcity of surgical services and safe anesthesia leads to acute, life-threatening complications and to chronic disabilities that hinder individuals from participating in gainful employment, placing burdens on their families and society.

Since the unanimous approval of the World Health Assembly Resolution on Emergency and Essential Surgery in 2015 (1), the scale-up to basic surgery in LMICs has been ongoing. Essential Surgery plays a vital role in addressing significant healthcare needs, cost-effectiveness, and feasibility in LMICs, with identified essential surgical procedures estimated to avert approximately 1.5 million deaths annually in LMICs (1, 3). These procedures rank among the most cost-effective health interventions, with investments in this area proving highly efficient. Strategies like task-sharing have expanded surgical access, but disparities in surgical care safety persist, necessitating measures like the World Health Organization's Surgical Safety Checklist.

Achieving Universal Health Coverage (UHC) and meeting the health-related targets outlined in the UN Sustainable Development Goals (SDGs) hinge on ensuring accessible, safe, timely, and affordable surgical, obstetrical, and anesthesia care. Traumatic injury alone causes more death and disability than HIV, malaria, and TB combined, making it a neglected global epidemic. Without prompt improvement in access to surgical care, LMICs stand to lose an estimated $12.3 trillion USD in economic output by 2030, highlighting the life-saving and economic growth potential of investing in surgical services.

Enhancing capacity in surgical care services in LMICs has a positive ripple effect on the entire health system, leading to improvements across all health sectors, patient age groups, and genders. However, currently, there is a lack of coordinated research or funding strategy to support the development of surgical care in LMICs, unlike other established mechanisms such as GAVI or the Global Fund for HIV, Malaria, and TB.

To improve patient safety in surgical care, especially anesthesia-related mortality and complications in LMICs, implementing the WHO Surgical Safety Checklist and quality improvement (QI) programs is essential. Developing standardized guidelines that integrate quality improvement, ethics, and legal dimensions is necessary for surgical missions to promote best practices and unify capacity building efforts (4).

Medical volunteerism plays a significant role in healthcare delivery, particularly in low-resource settings, but quality assurance (QA) processes and ethical considerations must be embedded into the missions to ensure effective and safe medical volunteerism. Cultural competence, power dynamics, sustainability, and potential harm are crucial ethical considerations in medical volunteerism, necessitating ethical guidelines, cultural sensitivity, and sustainable partnerships for successful surgical missions.

### Pediatric surgical care

Child health is of paramount importance in all countries, particularly in less developed nations where almost 50% of the population is under the age of 20, compared to the global average of 35% (5). While the spectrum of pediatric surgical conditions and their treatment may vary across nations, their relevance to recent advances in global surgery must be addressed. Though prioritizing surgical care within child health is ethical but also presents significant challenges. Global health initiatives should not solely focus on severe conditions such as heart diseases; rather, integration with surgical initiatives can achieve numerous important child health goals easily.

In low- and middle-income countries (LMICs), the critical needs of children's surgical care have been overlooked for far too long, despite the substantial pediatric population and increasing burden of surgical diseases (6). The scarcity of pediatric surgeons and limited infrastructure have exacerbated the problem, resulting in unacceptably high rates of morbidity, mortality, long-term disabilities, and economic strain on families. Nevertheless, the global initiative for children's surgery (GICS) has made significant strides in elevating the profile and visibility of children's surgery in the global health arena (6). GICS's approach, emphasizing inclusiveness, active involvement of LMICs, focus on their unique needs, and collaboration with high-income countries (HICs), has been instrumental in driving implementation and creating tangible change on the ground.

Efforts are being made to bolster infrastructure through the establishment of children's operating rooms, while national surgical plans are being gradually adapted to include children's surgery, providing a much-needed policy framework. In Nigeria, for instance, the pediatric surgery workforce has seen growth, yet the density remains low at 0.14 per 100,000 population under 15 years. To address knowledge gaps, education and training have been strengthened with the development of a pediatric surgery textbook for Africa and the creation of a Pan Africa pediatric surgery e-learning platform (7). However, it is noteworthy to state that financing children's surgery in LMICs remains a significant barrier, with many families at risk of facing catastrophic healthcare expenditure.

Global surgery, including global pediatric surgery, involves collaborative, cross-sectoral, and transnational approaches, combining population-based strategies with individual surgical care. Pediatric surgical conditions encompass various disease categories such as infections, injuries, cancer, and congenital anomalies, with a substantial burden arising from time-critical emergency conditions, potentially over 50%. These conditions can occur from neonatal to teenage years, and some require ongoing follow-up care throughout childhood. To ensure sustainability, it is crucial to establish local pediatric surgical capabilities with local leadership and ownership, aligning with the Ministry of Health's priorities and policies. Effective clinical leadership and early selection and support of multiple individuals for training are essential strategies for sustainability. Ensuring timely access to quality specialist advice, tailored to the population's needs, is the primary determinant of clinical outcomes (8).

### Short-term surgical missions

Short-term surgical missions involve healthcare providers from high-income countries traveling to low- and middle-income countries to provide surgical care. While these missions can be beneficial, they face challenges in terms of sustainability, follow-up care, and integration with existing healthcare systems. Surgical missions are more effective for prevalent conditions rather than surgical emergencies, and improving emergency treatment requires systemic improvements in healthcare capacity.

However, short-term missions have limitations (3). They are episodic and time-limited, which may restrict the number of patients treated and hinder training efforts. Outcomes vary based on surgical procedures, with higher mortality and complication rates associated with more complex procedures (9). Follow-up care and integration with local healthcare services may also be limited, making it difficult to meet comprehensive patient needs. Short-term missions are not designed for emergency life-threatening conditions, emphasizing the need for sustainable healthcare systems.

In summary, short-term surgical missions have advantages but also face challenges and produce mixed results. They are more suitable for simpler conditions rather than complex procedures. While cost-effective in certain contexts, their effectiveness and sustainability depend on various factors. Utilized alongside existing surgical delivery platforms, short-term missions can be a valuable and cost-effective method, but their stand-alone use should be approached with caution. With careful planning and adherence to best practices, short-term missions can positively impact healthcare settings and contribute to improved surgical outcomes.

### Quality improvement

Over the past two decades, there has been increasing attention given to the safety, quality, and cost of healthcare, leading to a greater emphasis on formal quality measurement and quality improvement (QI) programs. Quality improvement (QI) plays a vital role within the context of healthcare by facilitating systematic and continuous efforts to enhance the effectiveness, efficiency, safety, and patient-centeredness of healthcare services (10). This involves measuring performance, identifying areas for improvement, implementing evidence-based interventions, and evaluating their impact.

Assessment and measurement of QI interventions are crucial, and organizations like the American College of Surgeons’ National Surgical Quality Improvement Program (ACS-NSQIP) provide reliable data collection, feedback, and best practices initiatives. QI in surgery is a significant focus for surgeons, hospitals, professional organizations, insurers, and regulators, with efforts aimed at improving patient outcomes and care delivery. It is only understandable that quality assurance (QA) processes should improve the quality of care in developing countries, including medical volunteerism. While national governing bodies such as the American Board of Surgery and the Joint Commission mandate performance metrics monitoring and surgical safety, process improvement methodologies like DMAIC, Six Sigma, and Lean are also applied in surgical QI. Tools such as clinical mapping instruments, enhanced communication, and error reduction strategies support these efforts. However, these applications may face challenges when applied to QI in low- and middle-income countries.

Since QA processes are crucial for an effective and safe medical volunteerism, a well-established process must be sought of. For instance, pre-departure training, needs assessments, monitoring and evaluation, community engagement, recruitment and training of volunteers, and capacity building and sustainability efforts must be established.

Conducting needs assessments, monitoring, and evaluation, along with community engagement could be the only method to assure quality improvement and sustainability of medical volunteerism. The needs assessment may vary but should include a set of recommendations such as pre-departure training, in-country orientation, and evaluation of volunteer performance. These measures help ensure that medical volunteers are adequately prepared and capable of providing quality care within the available and affordable local resources. By ensuring that volunteers are competent and capable of providing quality care, the overall effectiveness of medical volunteerism can be enhanced. In other words, QA processes could be initially achieved prior to the mission and are pivotal in the recruitment and training of volunteers for global health volunteering. Since the purpose of medical missions is to leave behind a sustainable system, capacity building such as training and evaluation are mandatory (10). These processes enable volunteers to provide quality care and ensure that their efforts can be sustained over the long term.

It is important to select well-structured programs to optimize the experience of the host institution and the sending institution. A well-structured program should be one where there are clear delineation of roles and responsibilities, local needs are considered paramount, recognition of true cost to the host institution, and transparency in open communication between institutions (18). Periodic review of the programs by the sending institution, host institution, sponsors, and trainees help ensure that these programs continue to benefit all.

### Ethical concerns

Medical volunteerism raises several ethical concerns that require addressing, among which cultural competence stands out and has been emphasized in previous studies (11). It is important for medical volunteers to be aware of and respect the cultural norms and values of the communities they serve to ensure that their actions are culturally appropriate and sensitive.

Respecting the rights and dignity of the communities served is essential for the development of sustainable partnerships in global health clinical experience. In addition to cultural competence, ethical issues related to sustainability and potential harm in medical volunteerism must be considered. Working collaboratively with local communities and building sustainable partnerships are essential to ensure that medical volunteerism is conducted ethically and in line with local norms and values. Building sustainable partnerships fosters trust, facilitates knowledge exchange, empowers the local healthcare infrastructure, and promotes self-sufficiency within the community.

### Legal dimensions

Global health law encompasses various legal norms, processes, and institutions aimed at achieving the highest standard of health worldwide. While not centralized like the World Trade Organization, it relies on a network of treaties and “soft” law instruments, often developed under the guidance of the World Health Organization (WHO).

Medical volunteerism also involves legal considerations that vary depending on the country and context in which the work is being done. Understanding the legal framework, obtaining necessary documentation, and protecting against liability risks are crucial aspects that need to be addressed. Informed consent, liability, and licensure are among the legal aspects to be considered (12, 13).

Global health law operates without a centralized monitoring body, relying on binding treaties, customary international law, and non-binding guidelines. This framework enables countries to collaborate on infectious diseases, healthcare access, and health inequalities. It emphasizes legal mechanisms to address global health challenges, including principles, regulations, and agreements to protect and promote well-being worldwide. By setting standards, facilitating cooperation, and tackling cross-border health issues, global health law aims to improve health outcomes. Founded in 2002, The Children's Health International Medical Project of Seattle for example has outlined 7 guiding principles including mission, collaboration, education, service, teamwork, sustainability, and evaluation to serve as a model (14). The WHO plays a crucial role in shaping global health law. It develops international agreements, guidelines, and best practices to support optimal health for all. These initiatives foster global solidarity and address interconnected health challenges.

Healthcare professionals should familiarize themselves with the laws, regulations, and cultural practices surrounding informed consent in the host country. Adequate language skills or access to translators should be ensured to facilitate effective communication and culturally sensitive consent discussions with patients.

Liability is another important consideration. Healthcare professionals embarking on international medical volunteering must understand the legal framework that governs their activities in the host country (13). This entails comprehending their personal liability, understanding potential legal recourse for patients, and being aware of any limitations or protections provided by local laws or agreements with sponsoring organizations. It is essential to assess the extent of coverage offered by existing malpractice insurance policies and determine if it extends to international settings. Specialized insurance tailored for international medical work may be necessary to adequately safeguard both the healthcare professional and the patients they serve.

In brief, the rise of short-term global health missions (SMM), led by various organizations and involving participants such as students, healthcare professionals, and corporate leaders, requires clear ethical guidance to minimize harm and benefit host communities.

The lack of universal SMM guidelines has led to concerns about standardization and accountability. Host communities have voiced frustration over the negative impact on local healthcare systems and questioned volunteers' competence (15, 16).

To improve SMM, the Brocher Declaration promotes principles like mutual partnership, empowered host communities, sustainable programs, legal compliance, cultural sensitivity, and accountability (17). It encourages a shift from a “helping” to a “learning” and “sharing” approach.

While SMM is popular in high-income countries, it can be wasteful and harmful to host communities. In the absence of universal regulations, participants and organizations must uphold these principles and laws. This declaration calls for a reevaluation of volunteer-centric missions, aiming to remove barriers to health equity and foster shared responsibility for global health challenges.

## Conclusion

Surgical volunteerism remains important for improving global health, even as local surgical systems scale up, especially in low- and middle-income countries with limited access to specialized surgeries. Short-term missions offer much-needed expertise and resources for specialty surgical procedures, which may improve healthcare outcomes in children and adults who otherwise would not have access to care. To ensure quality and an ethical approach, local needs assessments, education and training, and ongoing outcomes monitoring are essential. Cultural competence, longitudinal commitment, and planning complications and poor outcomes are important for capacity building and ethical reassurance. Adhering to local licensure, credentialling and hospital approval must be addressed, and informed consent in the local language is expected. Maintaining high standards, consistent with best practice of the teams' home country, and a strict adherence to patient safety practice must be *a priori*ty for visiting surgical teams. Without these adherences and reassurances, the goals of visiting surgical teams cannot be achieved.
